# Cross-cultural adaptation, validity and reproducibility of the Back Beliefs Questionnaire among older Brazilians with acute low back pain. A cross-sectional study

**DOI:** 10.1590/1516-3180.2019.0542.R2.16042020

**Published:** 2020-07-03

**Authors:** Luiza Faria Teixeira, Juliano Bergamaschine Mata Diz, Silvia Lanziotti Azevedo da Silva, Joana Ude Viana, João Marcos Domingues Dias, Leani Souza Máximo Pereira, Rosângela Corrêa Dias

**Affiliations:** I PT. Doctoral Student, Department of Physical Therapy, Universidade do Vale do Sapucaí (UNIVAS), Pouso Alegre (MG), Brazil.; II PT. Master’s Student, Postgraduate Program on Rehabilitation Sciences, Department of Physical Therapy, Universidade Federal de Minas Gerais (UFMG), Belo Horizonte (MG), Brazil.; III PT, PhD. Professor, Department of Physical Therapy, School of Nursing, Universidade Federal de Alfenas (UNIFAL), Alfenas (MG), Brazil.; IV PT. Doctoral Student, Postgraduate Program on Rehabilitation Sciences, Department of Physical Therapy, Universidade Federal de Minas Gerais (UFMG), Belo Horizonte (MG), Brazil; V PT, PhD. Professor, Postgraduate Program on Rehabilitation Sciences, Department of Physical Therapy, Universidade Federal de Minas Gerais (UFMG), Belo Horizonte (MG), Brazil.; VI PT, PhD. Professor, Postgraduate Program on Rehabilitation Sciences, Department of Physical Therapy, Universidade Federal de Minas Gerais (UFMG), Belo Horizonte (MG), Brazil.; VII PT, PhD. Professor, Postgraduate Program on Rehabilitation Sciences, Department of Physical Therapy, Universidade Federal de Minas Gerais (UFMG), Belo Horizonte (MG), Brazil.

**Keywords:** Low back pain, Validation study, Reproducibility of results, Attitude, Aged, Back beliefs questionnaire, Validity, Reliability, Psychosocial factors, Older adults

## Abstract

**BACKGROUND:**

Low back pain (LBP) has emerging as an epidemic, multifactorial and multidimensional condition in older age. Assessment of attitudes and beliefs of patients with back pain is necessary for understanding the impact of psychosocial factors on pain perception and management.

**OBJECTIVES:**

To cross-culturally adapt and examine the validity and reproducibility (intra and interrater reliability and agreement) of the Back Beliefs Questionnaire (BBQ) in older Brazilians with acute LBP.

**DESIGN AND SETTING:**

Cross-sectional methodological report conducted at the Department of Physical Therapy of the Universidade Federal de Minas Gerais, Belo Horizonte, Brazil.

**METHODS:**

The present study was conducted for translating, adapting, and examining the psychometric properties of a questionnaire. Participants aged ≥ 60 years experiencing an acute episode of LBP were recruited. Coefficients of internal consistency, reliability and agreement were obtained using Cronbach’s α, intraclass correlations, and standard error of measurement and the smallest detectable change, respectively.

**RESULTS:**

Twenty-six participants aged between 60-84 years and reporting a mean of 9.8 (4.3) years of schooling completed the study. The Brazilian Portuguese-language version of the BBQ (BBQ-Brazil) was proposed and presented with adequate conceptual, semantic, operational, and measurement equivalence from the original version. Intra and interrater evaluations showed moderate (0.74) and excellent (0.91) intraclass correlation coefficients, respectively, with small standard error of measurement for both evaluations. Internal consistency was considered adequate (0.70).

**CONCLUSION:**

BBQ-Brazil had consistent measurements of validity and reproducibility, and proved to be a valuable tool in clinical practice for addressing attitudes and beliefs of older patients with acute LBP.

## INTRODUCTION

Low back pain (LBP) has been a challenge for gerontology in relation to promoting healthy aging. Population-based surveys have attested that LBP gives rise to enormous epidemiological, clinical and economic burdens in the older population, with high prevalence and disability rates worldwide.^1^^,^^2^ A recent systematic review showed that one in four Brazilians aged ≥ 60 years was suffering from LBP at any given moment.^3^ More importantly, longitudinal studies have shown that about 40% of older individuals do not recover within 12 months of pain onset.^4^ Their pain may evolve towards chronic pain, thus leading to severe functional impairment, social deprivation, depression and permanent incapacity.^1^^,^^5^

The transition from acute to persistent LBP among older patients can be explained in terms of psychosocial factors that significantly influence their functional status.^6^ Psychosocial factors known as “yellow flags” increase the risk of long-term disability, and early screening for these factors is needed in order to prevent chronic LBP. In this regard, patients’ attitudes and beliefs about pain should be highlighted.^7^ The domains of these factors result from customs, ideologies, values and religious, and spiritual experiences, and they influencing individual behavior and social life at all levels, from interpersonal to political, economic and legal relationships.^8^ Negative beliefs are associated with poor recovery among older adults, after an acute episode of LBP.^6^^,^^7^

The Back Beliefs Questionnaire (BBQ) was developed by Symonds et al.^8^ to assess attitudes and beliefs among patients with back pain and those for whom future back problems are unavoidable. It was previously shown to have high validity/reliability coefficients (e.g. internal consistency ≥ 0.70 and test-retest reliability ≥ 0.80) when used for the general population in clinical settings.^8^^,^^9^^,^^10^^,^^11^ However, even though LBP has now emerged as an epidemic, multifactorial and multidimensional condition in older age,^1^^,^^2^^,^^6^^,^^7^ no study has, to the best of the authors’ knowledge, investigated the psychometric properties of the BBQ among older adults with acute back complaints.

## OBJECTIVE

The purpose of the present study was to cross-culturally adapt and examine the validity and reproducibility (intra and interrater reliability and agreement) of the BBQ in older Brazilians with acute LBP.

## METHODS

### Study design and participants

This was a methodological report using data from a subsample of the Brazilian cohort “Back Complaints in the Elders” (BACE-Brazil). This formed part of an international multicenter study including Brazil, the Netherlands and Australia that was designed to investigate the clinical course and prognostic factors of LBP among older individuals. The BACE protocol has been published in detail elsewhere.^12^

For this report, a convenience sample of 42 individuals was recruited through advertisements in local newspapers, radio and the internet, and through active searching or referrals from healthcare professionals at public and private primary care services. BACE-Brazil was approved by the local ethics committee on February 24, 2016, under the approval number ETIC 0100.0.203.000-11, and all participants signed an informed consent form.

The inclusion criteria were that the participants needed to be community-dwelling people aged ≥ 60 years who presented with a new (acute) episode of LBP, i.e. any pain between the lower ribs and inferior gluteal folds, with or without leg symptoms, which had occurred for a period shorter than six weeks. An episode of LBP was considered new if the participant had not sought medical care due to this condition during the preceding six months before the time of data collection.^12^

The exclusion criteria were the presence of severe diseases (e.g. infectious diseases, malignant tumors and cauda equina syndrome); severe visual, hearing, or motor loss; and cognitive impairment detectable through the Mini-Mental State Examination (MMSE), using the Brazilian cutoff points according to schooling level, as follows: 13 for illiterate individuals, 18 for those with < eight years of schooling, and 26 for those with ≥ eight years of schooling.^13^

### Instruments and measurements

**Figure 1.**
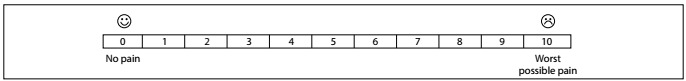
A model of the 11-point visual numerical rating scale that was used to assess pain intensity in the present study.

Sociodemographic and clinical data were collected by trained researchers using a standardized multidimensional questionnaire that included age (years), sex (female/male), schooling (years), pain intensity (0-10) and “sought medical care due to LBP over the past 6 weeks?” (yes/no). Pain intensity was assessed using an 11-point visual numerical rating scale ranging from 0 (“no pain”) to 10 (“worst possible pain”) (Figure 1). The question on pain intensity was asked in relation to two times: “at the present time” of data collection and “over the past week” before data collection.

The BBQ is composed of nine statements, including five questions that are used as distractors, totaling 14 items. Respondents report their level of agreement on a Likert scale ranging from 1 (completely agree) to 5 (completely disagree). The total score is calculated by inverting the sum of scores from the 9 affirmations and can range from 9 to 45 points. The smaller the score is, the more negative the respondent’s attitudes and beliefs are.^8^ The BBQ exhibits good internal consistency and reliability estimates, with Cronbach’s α between 0.70 and 0.81 and intraclass correlation coefficient (ICC) between 0.80 and 0.87, respectively.^8^^,^^9^^,^^10^^,^^11^

### Translation and cross-cultural adaptation

Language adaptation was performed in five steps: (i) conceptual equivalence: presentation of the same concepts; (ii) item equivalence: adjustment of elements from the original scale to represent the concepts of the language in question; (iii) semantic equivalence: transfer of meaning from one language to the other; (iv) operational equivalence: checking the possibility of using a similar format of questionnaire, instructions and application form; and (v) measurement equivalence: examining whether the different versions of the questionnaire reach similar levels of validity and reliability as in the original questionnaire.^14^

Semantic equivalence was assessed in accordance with the stages proposed by Beaton et al.:^15^ (1) translation: the instrument was translated to the Brazilian Portuguese language by two independent professional translators (T1 and T2) who were both native speakers of Brazilian Portuguese and proficient in the English language; (2) translation synthesis: a third person arranged a final version (T12); (3) back-translation: another two individuals (R1 and R2), who were independent from the previous two translators, back-translated the T12 version into the English language without having had any contact with the original version; (4) experts’ analysis: a committee of experts in the field (e.g. physicians, physiotherapists and occupational therapists) was created to review the final version; and (5) pre-testing: application of the pre-final version (pilot test) to a sample of older persons, to assess the comprehension and adequacy of the final questionnaire.

### Statistical analysis

The participants’ characteristics were described using means (with standard deviation) for numerical variables and absolute numbers (with percentage) for categorical variables. The normality of numerical data distribution was tested using the Kolmogorov-Smirnov test. Internal consistency was assessed by means of interrater measurements using Cronbach’s α coefficient. A value of α ≥ 0.70 was considered to be an acceptable level of internal consistency.^16^

The intrarater (test-retest) reliability was estimated using ICC type 3.1 with a two-way mixed-effects model.^17^ The consistency of each examiner’s measurement (model 3) was evaluated in duplicate during the comparison of single measurements. For the intrarater assessment, the BBQ was applied twice to the same participant by the same examiner, within an interval of 7 to 14 days.^15^

The interrater reliability was estimated using ICC type 2.1 with a two-way random-effects model.^17^ Two different examiners (model 2) were evaluated by simple comparison of two measurements. For the interrater assessment, the BBQ was applied by two examiners to one participant on the same day.^15^ The ICC was classified as poor (< 0.40), moderate (between 0.40 and 0.75), substantial (between 0.75 and 0.90) or excellent (> 0.90).^16^ All questionnaires were applied under supervision to avoid bias in cases of subjects with poor educational level.

Agreement was assessed using the standard error of measurement (SEM) and the smallest detectable change (SDC), which were calculated for the intra and interrater reliability coefficients. The SEM was calculated as the ratio between the standard deviation of the mean difference and two squared. The SDC was calculated using the following formula: SDC = 1.96 x √2 x SEM.^16^ Lower SEM and SDC values indicated less error and higher concordance between measurements. The dispersion of the results from both measurements was examined through agreement analysis using Bland-Altman plots, and it was checked whether the intra and interrater estimates were encompassed within the “agreement limits” (established as 1.96 times the standard deviation of the measurements).^18^

Significant differences were inferred to exist at the level of a two-tailed P < 0.05. All the analyses were conducted using the SPSS software package, version 18.0 (SPSS Inc., Chicago, IL, USA).

## RESULTS

### Sample characterization

The participants’ descriptive data are presented in Table 1. Twenty-six of the 42 individuals recruited (who were all aged between 60 and 84 years) attended both the first and the second time-points of data collection, and their data were analyzed. The other 16 individuals were excluded from the analysis because they did not complete the entire data collection process. Most of the participants included were female (88.5%), had a medium-to-high schooling level (≥ 9 years), reported moderate pain intensity (scoring between 4 and 7 on the 0-10 scale), and had not sought medical care due to LBP over the past 6 weeks (69.2%).

**Table 1.** Descriptive sample characteristics (n = 26)

Variables	Mean (SD) or n (%)
Age (years)	67.4 (5.8)
Female	23 (88.5%)
Schooling (years)	9.8 (4.3)
Pain intensity “at the present time” (0-10)	5.0 (3.1)
Pain intensity “over the past week” (0-10)	7.4 (2.2)
Sought medical care due to LBP^1^ (yes)	8 (30.8%)

^1^Variable referring to “sought medical care due to LBP over the past six weeks?” (yes/no).

SD = standard deviation; LBP = low back pain.

### BBQ adaptation, validity and reproducibility

#### Conceptual and item equivalence

The expression “attitudes and beliefs about LBP” was universally accepted as reviewed in the literature, thus showing that there was conceptual equivalence between the English and Brazilian Portuguese languages. Other terms among the BBQ questions were also adequate for different cultures and countries, i.e. these items showed equivalence. The title of the questionnaire was kept as in the original English-language version, in order to preserve the internationally used language: *Brazilian Portuguese version of the Back Beliefs Questionnaire* (BBQ-Brazil).

The term “back trouble” (“back problem”) was translated and replaced by “back pain”. The original author of the BBQ was contacted about this, and it was clarified that this term could also refer to back pain. Indeed, the term “back pain” is very often used in Brazil, regardless of sociocultural factors such as age group, schooling and region of the country, and thus might correspond more accurately to the presence of LBP.

#### Semantic equivalence

After a critical review, the experts’ committee decided on the best meanings and arrangements of the BBQ items. A literal translation was possible, except in relation to the following expressions.

In item 2, “Back trouble will eventually stop you from working”, the expression “stop you from working” was translated into “*make you stop working*”, and so “*Back pain will eventually make you stop working*”. In item 5, *“*bad back should be exercised*”*, the question was adapted into “*A bad back should be exercised (for the back)*”. In item 10, “Back trouble means long periods of time off work”, the expression “time off work” makes the meaning more specific indicating that it is a time of absence from work due to pain and not any time different from work time, and so this question in Brazilian Portuguese is “*Back pain means long periods of time out off work*”*.*

In item 12, “Once you have had back trouble there is always a weakness”, it was suggested the addition of the term “*difficulty*” in the end of the question, which was used as an anchor for a better understanding of the expression “weakness”; this word was substituted by the expression “*weak point*” to make it clear that it is not a matter of muscle weakness, but a condition of vulnerability, concluding: “*Once you have had back pain there is always a weak point (difficulty)*”. In item 13, “Back trouble must be rested”, the word “*needs*” (instead of “must”) in Brazilian Portuguese was the one that best expressed this question: “*Back pain needs to be rested*”. Lastly, in item 14, “Later in life back trouble gets progressively worse”, the expression “later in life” was adapted to “*with aging*”, which indicates the same condition: “*With aging back pain gets progressively worse*”. An examiner’s manual addressing the application of the BBQ-Brazil was proposed and used during data collection (Appendix 1).

#### Operational equivalence

The BBQ-Brazil was applied to 26 participants, who exhibited adequate comprehension of the items (Appendix 1). Therefore, there was no need for a new experts’ committee meeting.

#### Measurement equivalence

**Figure 2.**
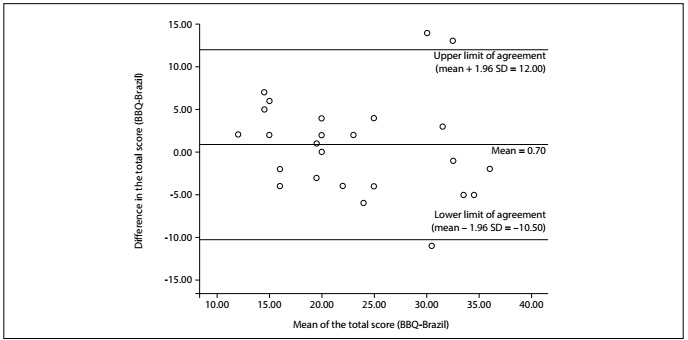
Bland-Altman plot for intrarater measurements demonstrating the mean differences ± 1.96 standard deviation (SD) limits of agreement using the Brazilian Portuguese version of the Back Beliefs Questionnaire (n = 26).

**Figure 3.**
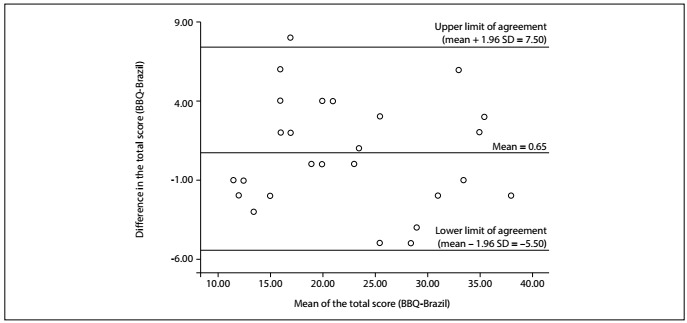
Bland-Altman plot for interrater measurements demonstrating the mean differences ± 1.96 standard deviation (SD) limits of agreement using the Brazilian Portuguese version of the Back Beliefs Questionnaire (n = 26).

Intra and interrater assessments revealed, respectively, moderate (ICC = 0.74) and excellent (ICC = 0.91) reliability coefficients for the BBQ-Brazil. Likewise, agreement estimates only showed small error between intrarater measurements (SEM = 4.03 and SDC = 11.05) and interrater measurements (SEM = 2.44 and SDC = 6.74) (Table 2). The Bland-Altman limits of agreement ranged from -10.50 to 12.00 for intrarater measurements (Figure 2), and from -5.50 to 7.50 for interrater measures (Figure 3). The internal consistency of the adapted questionnaire was adequate (Cronbach’s α coefficient = 0.70).

**Table 2.** Intra and interrater reliability and agreement results from the Brazilian Portuguese version of the Back Beliefs Questionnaire (n = 26)

Examiner	1^st^ measurement Mean (SD)	2^nd^ measurement Mean (SD)	ICC	95% CI	SEM	SDC
1	23.81 (7.46)	22.92 (8.16)	0.74	0.49 to 0.87	4.03	11.05
2	-	22.27 (8.44)	0.91	0.81 to 0.96	2.44	6.74

SD = standard deviation; ICC = intraclass correlation coefficient; 95% CI = 95% confidence interval; SEM = standard error of measurement; SDC = smallest detectable change.

## DISCUSSION

BBQ was translated, adapted, and tested in a Brazilian Portuguese-language version for older adults with acute LBP. The new questionnaire exhibited satisfactory performance regarding cross-cultural equivalence and psychometric properties and may be clinically useful for assessing the psychosocial behavior of older patients facing back pain symptoms. The original BBQ in the English-language version includes adaptation to a great number of different cultures.^8^ Other methodological studies conducted on general populations in Canada,^11^ Australia, Singapore and Taiwan,^10^ and China^19^ attest to the construct validity of this tool.

The original version was validated using a sample of workers from a biscuit factory located in northern England, where 70% of the sample were less than 45 years old.^8^ The study including participants from Australia, Singapore and Taiwan was conducted among physiotherapy and nursing students of mean ages 20.3 (1.3) and 20.5 (1.0) years, respectively.^10^ The study with the Chinese sample was conducted among healthcare professionals (i.e. physiotherapists, osteopaths and nurses) with a mean age of 40.3 (11.1) years.^19^

On the other hand, the present study was performed using a sample of older participants of mean age 67.4 (5.8) years and mean schooling of 9.8 (4.3) years. People of advanced age and lower schooling level tend to present poorer health outcomes and more negative complaints about back pain that impacted on their activities of daily living, while younger persons tend to have more positive beliefs regarding back pain, which thus led to better functional status.^6^^,^^7^ Likewise, lower schooling reduces the rate of seeking healthcare and is associated with negative beliefs and lower functional performance.^8^^,^^10^^,^^19^

Pain intensity “at the present time” of data collection was moderate, since most participants scored between 4 and 7 on the 0-10 scale, but pain intensity “over the past week” before data collection was moderate to severe (i.e. score > 7 on the 0-10 scale). Burnett et al.^10^ found mild pain among physiotherapist and nursing students, whereas the Chinese sample reported mild pain while resting and in the last week, moderate pain intensity in the last acute pain episode, and severe pain intensity in the worst pain episode.^19^

The majority of the participants did not seek healthcare services to treat LBP. This can be explained by difficulties in accessing healthcare services, low levels of physical capacity, attitudes of waiting for spontaneous recovery from pain, self-medication, use of resting or lack of interest because of repetitive pain episodes.^20^ In addition, unpreparedness among healthcare professionals still exists with regard to dealing with psychosocial outcomes such as negative attitudes and beliefs. The focus is only on physical symptoms that tend to persist, which leads to demotivation among patients in relation to the need for LBP treatment to be continuous.^7^

The mean BBQ score among these older Brazilians indicated that they had negative attitudes and beliefs, compared with the means from the Australian^10^ and Canadian^11^ studies, which were higher. Assessments made during the crisis period may have contributed to reports of negative attitudes and beliefs in relation to LBP. In the original validity study, the mean BBQ score tended to be more positive among the office workers than among the factory workers.^8^ Burnett et al.^10^ found positive beliefs among the subjects in Australia and Taiwan, and the participants in the Chinese study also showed positive attitudes and beliefs, in two evaluations.^19^

The BBQ was originally developed to assess attitudes and beliefs about back problems that caused absenteeism.^8^ However, it is important to validate and assess the applicability of this questionnaire in different populations and cultures. The process of cross-cultural validation among Brazilians and in other populations^10^^,^^11^^,^^19^ was based on similar standardized proposals and on consolidated references.^15^^,^^16^^,^^21^

During the cross-cultural adaptation to the Brazilian Portuguese language, the concept of “back problem” was adapted through cultural-semantic analysis into “back pain” because of the similarity between these concepts, as used in the Brazilian study population. In the Chinese version of the BBQ, few participants had difficulties in understanding the expression “back problem” and just a brief explanation of the term “back pain” was included.^19^

Semantic adaptation was necessary in some questions of the BBQ-Brazil and in other versions of the BBQ. For example, in the Chinese version, item 10 (“Back trouble means long periods of time off work”) was adapted to mean formal or paid work.^19^ Differently, in Brazil, a number of older people still continue to do formal work even after retirement, and many older people who have retired still working informally and/or at home. Because of this wide diversity of work possibilities, item 10 was kept and adapted according to the participants’ need during application of the questionnaire. Another question that required to semantic adaptation, which was highlighted both in the Brazilian and in the Chinese^19^ versions was item 12: “Once you have had back trouble there is always a weakness”. Some Brazilian and Chinese^19^ participants understood the word “weakness” as physical disability in general. Thus, in these both languages, “weakness” was replaced by “weak point” or “difficulty”, because the authors considered these expressions to be more appropriate for preserving cultural equivalence.

The assessment of measurement equivalence showed that the test-retest (ICC = 0.74) and interrater (ICC = 0.91) coefficients for the BBQ-Brazil were at adequate levels. The time interval between questionnaire applications regarding test-retest measurements may have compromised the similarity between responses because fluctuations in pain intensity can differentiate attitudes and beliefs about LBP. In contrast, the application between examiners was done on the same day, which will have reduced the influence of changes in pain perception and patients’ beliefs. No clinical changes in pain levels relating to the timing of questionnaire application were observed in other studies,^10^^,^^19^ and this was probably because they did not include participants’ reports of the acuteness of their pain. Lastly, Cronbach’s α = 0.70 indicates acceptable internal consistency, meaning that the questionnaire items measured the same construct and provided similar results between examiners.

Other instruments that are used for investigating psychosocial factors among patients with LBP had previously been adapted and validated for use in the Brazilian Portuguese language. Lopes et al.^22^ adapted the Pain Catastrophizing Scale for older Brazilians with acute LBP and found substantial coefficients of reliability (ICC = 0.88) and internal consistency (Rasch analysis = 0.95). Abreu et al.^23^ adapted the Fear Avoidance Beliefs Questionnaire for Brazilians aged 20 to 75 years with chronic LBP and also reported excellent coefficients of reliability (ICC = 0.91) and internal consistency (Cronbach’s α = 0.90). Although these instruments were used in different contexts of back pain, it may be interesting to combine them with the BBQ-Brazil, in order to obtain additional information about the psychological expectations and experiences of older patients with back complaints. The use of such instruments should be encouraged both in clinical and in research settings.^6^^,^^24^

The present study had certain strengths and limitations. The BBQ was translated, adapted and validated for use among older Brazilians with acute LBP through rigorous methodological approaches that included carefully applied face-to-face interviews with elderly people in order to control for the influence of low schooling levels. Therefore, the BBQ-Brazil might help healthcare professionals to manage LBP and thus reduce the epidemiological and clinical burden of this condition in the older population of Brazil. On the other hand, there was great difficulty in recruiting participants in accordance with the inclusion and exclusion criteria of this study, which meant that it was only possible to include a convenience sample with small number of participants (among whom 88.5% were women), thereby limiting the generalizability of the results.

## CONCLUSION

The BBQ was successfully translated and adapted for use among older Brazilians with acute LBP. Good validity/reproducibility coefficients were obtained for the BBQ-Brazil using intra and interrater measurements. The attitudes and beliefs of patients with back pain are important factors regarding the development of disabling chronic pain. They relate to coping behavior and treatment expectations, which can be positively modified through public health strategies. Psychosocial screening is essential, in order to encourage healthcare professionals to motivate their older patients to have an active life, avoid immobility and maintain independence and autonomy.

## References

[1] Stewart Williams J, Ng N, Peltzer K (2015). Risk Factors and Disability Associated with Low Back Pain in Older Adults in Low- and Middle-Income Countries. Results from the WHO Study on Global AGEing and Adult Health (SAGE). PLoS One.

[2] Depintor JD, Bracher ES, Cabral DM, Eluf-Neto J (2016). Prevalence of chronic spinal pain and identification of associated factors in a sample of the population of São Paulo, Brazil: cross-sectional study. Sao Paulo Med J.

[3] Leopoldino AA, Diz JB, Martins VT (2016). Prevalence of low back pain in older Brazilians: a systematic review with meta-analysis. Rev Bras Reumatol Engl Ed.

[4] Scheele J, Luijsterburg PA, Bierma-Zeinstra SM, Koes BW (2012). Course of back complaints in older adults: a systematic literature review. Eur J Phys Rehabil Med.

[5] Prince MJ, Wu F, Guo Y (2015). The burden of disease in older people and implications for health policy and practice. Lancet.

[6] Scheele J, Enthoven WT, Bierma-Zeinstra SM (2013). Course and prognosis of older back pain patients in general practice: a prospective cohort study. Pain.

[7] Wong AY, Karppinen J, Samartzis D (2017). Low back pain in older adults: risk factors, management options and future directions. Scoliosis Spinal Disord.

[8] Symonds TL, Burton AK, Tillotson KM, Main CJ (1996). Do attitudes and beliefs influence work loss due to low back trouble?. Occup Med (Lond).

[9] Maki D, Rajab E, Watson PJ, Critchley DJ (2017). Translation, cross-cultural adaptation and psychometric properties of the Back Beliefs Questionnaire in Modern Standard Arabic. Disabil Rehabil.

[10] Burnett A, Sze CC, Tam SM (2009). A Cross-cultural Study of the Back Pain Beliefs of Female Undergraduate Healthcare Students. Clin J Pain.

[11] Bostick GP, Schopflocher D, Gross DP (2013). Validity evidence for the back beliefs questionnaire in the general population. Eur J Pain.

[12] Scheele J, Luijsterburg PA, Ferreira ML (2011). Back complaints in the elders (BACE); design of cohort studies in primary care: an international consortium. BMC Musculoskelet Disord.

[13] Bertolucci PHF, Brucki SMD, Campacci SR, Juliano Y (1994). O Mini-Exame do Estado Mental em uma população geral: impacto da escolaridade [The Mini-Mental State Examination in an outpatient population: influence of literacy]. Arq Neuro-Psiquiatr.

[14] Herdman M, Fox-Rushby J, Badia X (1998). A model of equivalence in the cultural adaptation of HRQoL instruments: the universalist approach. Qual Life Res.

[15] Beaton DE, Bombardier C, Guillemin F, Ferraz MB (2000). Guidelines for the process of cross-cultural adaptation of self-report measures. Spine (Phila Pa 1976).

[16] Terwee CB, Bot SD, de Boer MR (2007). Quality criteria were proposed for measurement properties of health status questionnaires. J Clin Epidemiol.

[17] Shrout PE, Fleiss JL (1979). Intraclass correlations: uses in assessing rater reliability. Psychol Bull.

[18] Bland JM, Altman DG (1986). Statistical methods for assessing agreement between two methods of clinical measurement. Lancet.

[19] Chen G, Tan BK, Jia HL, O’Sullivan P, Burnett A (2011). Questionnaires to examine Back Pain Beliefs held by health care professionals: a psychometric evaluation of Simplified Chinese versions. Spine (Phila Pa 1976).

[20] Docking RE, Fleming J, Brayne C (2011). Epidemiology of back pain in older adults: prevalence and risk factors for back pain onset. Rheumatology (Oxford).

[21] Eremenco SL, Cella D, Arnold BJ (2005). A comprehensive method for the translation and cross-cultural validation of health status questionnaires. Eval Health Prof.

[22] Lopes RA, Dias RC, Queiroz BZ (2015). Psychometric properties of the Brazilian version of the Pain Catastrophizing Scale for acute low back pain. Arq Neuropsiquiatr.

[23] Abreu AM, Faria CD, Cardoso SM, Teixeira-Salmela LF (2008). Versão brasileira do Fear Avoidance Beliefs Questionnaire [The Brazilian version of the Fear Avoidance Beliefs Questionnaire]. Cad Saude Publica.

[24] Silva JPD, Jesus-Moraleida F, Felicio DC (2019). Biopsychosocial factors associated with disability in older adults with acute low back pain: BACE-Brazil study. Cien Saude Colet.

